# Outcome of post‐prostatectomy stress urinary incontinence surgery in men with preoperative idiopathic detrusor overactivity

**DOI:** 10.1002/bco2.442

**Published:** 2024-10-03

**Authors:** Nikita R. Bhatt, Simona Ippoliti, Arjun Nambiar, Cristian Ilie, Ruth Doherty, Lee Smith

**Affiliations:** ^1^ Department of Urology Norfolk and Norwich University Hospitals Norwich UK; ^2^ Department of Urology Hull University Teaching Hospitals Hull UK; ^3^ Department of Urology, Newcastle upon Tyne NHS Foundation Trust Newcastle UK; ^4^ Centre for Health, Performance and Wellbeing Anglia Ruskin University Cambridge UK

**Keywords:** male incontinence, overactive bladder, radical prostatectomy, storage dysfunction, stress urinary incontinence

## Abstract

**Background:**

Urodynamic evidence of storage dysfunction such as detrusor overactivity (DO) and/or poor compliance are present in up to 30–40% of patients after Radical Prostatectomy (RP). However, the current optimal management of men with DO on preoperative urodynamics prior to male stress urinary incontinence (SUI) surgery is not known.

**Methods:**

We performed a systematic search of the literature including articles on patients undergoing SUI surgery after prostatectomy with preoperative DO between January 2003 and May 2023 to ensure contemporaneous data was obtained.

**Results:**

We identified 11 eligible publications with a total of 792 patients. On Urodynamics, 29% (n = 229) patients had DO prior to SUI surgery. Overall 69% patients had a successful outcome after SUI surgery while 26% (132/499) failed while 34% (32/95) patients who had proven DO preoperatively failed SUI surgery. The difference was not statistically significant. Considering the sub‐group analysis, the failure rate with preoperative DO was significantly higher in the sling group (43%) than in the AUS group (18%). The review was limited by the outcome heterogeneity, variability in study inclusion criteria, reporting and analysis and the quality of the available studies.

**Conclusions:**

Within the limitations of the data, this review did not show a statistically significant higher failure rate of male incontinence surgery in patients with DO. Hence, patients with DO on preoperative urodynamics who are eligible for male SUI surgery should not be denied surgery but should be counselled appropriately of the risks and potential need for subsequent treatment, to manage expectations.

**Lay summary**

In the context of male incontinence surgery, more than a third of patients experience a condition known as detrusor overactivity. This condition involves involuntary bladder contractions, which can lead to sudden urges to urinate and, in some cases, urinary leakage. However, there is ongoing confusion regarding how to define success and failure in these surgeries, particularly when detrusor overactivity is present. To address this, high‐quality studies with well‐defined criteria are necessary. Until such evidence is available, it is important to counsel patients with detrusor overactivity rather than excluding them from surgery based on the current lack of conclusive evidence.

## 1 INTRODUCTION

Urinary incontinence after radical prostatectomy (RP) is a recognised complication. Postprostatectomy Stress Urinary Incontinence (PPI‐SUI) is present in 2.5–90% of patients after RP depending on the definition and the follow‐up period.^1^ PPI‐SUI is a multi‐factorial, complex entity resulting from a combination of factors such as injury to the external rhabdosphincter, internal sphincter deficiency and underactivity, neural impairment, urethral support defects, decreased membranous urethral length and venous sealing effect.^1^, ^2^ Other risks factors include advanced age, obesity, preoperative bladder dysfunction, prostate volume, patient comorbidities and previous benign surgery.^1^, ^2^ PPI‐SUI is problematic as it is associated with several adverse outcomes such as negative psychological impact, increased risk of falls, fragility and pressure ulcers.^3^ Urodynamic evidence of storage dysfunction such as detrusor overactivity (DO) and/or poor compliance are present in up to 30–40% of these patients and may contribute to PPI‐SUI.^1^, ^2^ However, the impact of this storage dysfunction on symptoms and management of PPI‐SUI is not fully understood.

The incidence of Overactive Bladder (OAB) in the general population is approximately 11% and is age‐dependent.^4^ Historical reviews suggest de‐novo urodynamic DO rates following RP in the range of 2–77% when assessed 6 months after RP, persisting in the majority of patients (83%).^5^ It is commonly associated with other forms of voiding dysfunction (including reduced bladder compliance and stress urinary incontinence [SUI]), but this review does not report the proportion of patients with Urodynamic DO who were symptomatic with OAB. A review by Thiruchelvam et al^6^ outlined a wide variation in the severity of OAB and its role in bothersome lower urinary tract symptoms (LUTS) after radical prostate cancer treatment. Theories on the development of OAB symptoms include partial bladder decentralisation both centrally and peripherally due to mobilisation during the RP, combined with somatic denervation; inflammation or infection and geometric bladder wall alteration associated with pre‐existing hypoxemia with or without neuroplasticity. They reported that OAB occurs more frequently after radiotherapy in the early phase but improves rapidly, with the combination of brachytherapy and external beam radiotherapy (EBRT) resulting in the most severe and long‐lasting symptoms.^6^ In addition, men receiving further treatment with adjuvant or salvage radiotherapy after RP have a statistically significant increase in the risk of developing OAB.^4^

Pure SUI after RP can be adequately treated by further surgery. However, OAB symptoms after prostate cancer treatment can be more difficult to treat. Based on their review, Thiruchelvam^6^ and colleagues suggest not categorically denying men with SUI and coexistent OAB male incontinence surgery but suggested making a judgement based on urodynamics in each case with careful patient counselling. Considering almost a third of patients^7^ with PPI have an element of DO contributing to their incontinence, understanding the impact of preoperative DO on male incontinence surgery outcomes is vital for counselling and consenting patients. In addition, it is also imperative for clinicians managing PPI‐SUI to understand the outcomes of patients undergoing male incontinence surgery on a background of pre‐existing DO to enable careful patient selection. Despite this, no attempt has yet been made to collate the literature on these topics, necessary to derive a precise understanding. With this in mind, our aim was to perform a systematic review of the outcomes of patients undergoing stress incontinence surgery after prostatectomy with preoperative co‐existing detrusor overactivity.

## 2 EVIDENCE ACQUISITION

### 2.1 Search strategy

**FIGURE 1:**
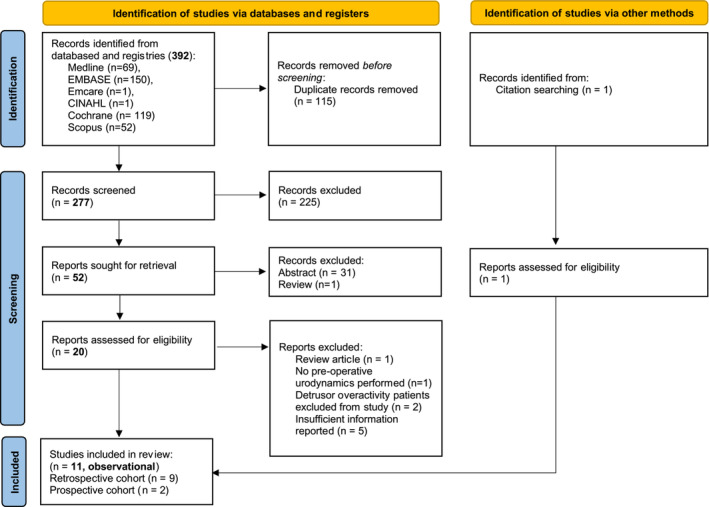
PRISMA flowchart of studies.Page MJ, McKenzie JE, Bossuyt PM, Boutron I, Hoffmann TC, Mulrow CD, et al. The PRISMA 2020 statement: an updated guideline for reporting systematic reviews. BMJ 2021;372:n71. Doi: 10.1136/bmj.n71. For more information, visit: http://www.prisma‐statement.org/

The study protocol was registered on PROSPERO (CRD42023395830) prior to undertaking a systematic search of the literature using MEDLINE/PubMed, Embase, Emcare, Web of Science and Cochrane databases. A manual search of the reference lists of any relevant systematic reviews was also conducted. The full search strategy is provided in Appendix A. The results are reported using the Preferred Reported Items for Systematic Reviews and Meta‐analyses (PRISMA) statement (Figure 1).^8^

### 2.2 Study selection, inclusion and exclusion criteria

Full‐text studies, including both randomised controlled trials (RCTs) and observational studies conducted between January 2003 to May 2023 were selected for inclusion to ensure contemporaneous data was obtained. Conference abstracts, reviews, editorials, research letters and comments were excluded. We included articles published in English only, on humans only, on male patients and across all age groups, and on patients undergoing stress incontinence surgery after prostatectomy with preoperative urodynamic detrusor overactivity. Studies including newer procedures or less established procedures (e.g.: ProACT) and studies lacking postoperative outcome data (e.g.: continence rates) were excluded.

Two authors (NB and SI) independently performed the initial screening of all the available studies and identified the final studies for inclusion. There was 100% agreement between the reviewers.

### 2.3 Data extraction

Two authors (NB and SI) independently performed data extraction, both across all studies, and any discrepancies were resolved by a third author (AN) after discussion. We included study characteristics such as author, year of publication, country, study type, number of patients included and number of patients with preoperative DO. We included details of incontinence surgery including the type of procedure, a prosthetic device used, the definition of success and the proportion of patients with a successful outcome. Details of the subgroup (patients with preoperative DO prior to male incontinence surgery) being studied were included such as success and failure definitions as well as rates.

### 2.4 Statistical analysis

Mean and standard deviation (SD) or medians and interquartile range were selected for continuous variables as appropriate. For dichotomous variables, the number of events as a proportion of the sample size was collected. Pooled estimates were obtained using means and SDs for continuous variables while event rates were obtained for dichotomous variables. The presence of more than one RCT per outcome qualified for meta‐analysis. For studies with multiple publications, only the most up‐to‐date or complete data for each outcome was utilised. Quantitative synthesis was not undertaken for non‐randomised studies. A priori, a fixed effects model was used to calculate pooled estimates of treatment effect across similar studies and their 95% CIs. If clinical or methodological heterogeneity was expected, then a random effects model was used. For time‐to‐event data, the log (hazard ratio) and its variance were combined using the generic inverse variance method. Dichotomous outcomes were combined using the Mantel–Haenszel method for risk ratios or odds ratios. Continuous outcomes were combined using the inverse variance mean difference method. If studies used different scales to assess the same continuous outcome, the standardised mean difference was preferred over the mean difference. If meta‐analyses were deemed inappropriate, a narrative synthesis approach was used to summarise the results.

### 2.5 Risk‐of‐bias assessment

Risk of bias assessment was undertaken using the Risk of Bias in non‐Randomised Studies of Interventions (ROBINS‐1) tool, independently by two authors (NB and SI). The Risk of Bias (RoB2) revised Cochrane tool was used for RCTs. Any disagreements were resolved by a third author (AN).

## 3 EVIDENCE SYNTHESIS

### 3.1 Study characteristics

We identified 11 eligible publications with a total of 792 patients,^9^, ^10^, ^11^, ^12^, ^13^, ^14^, ^15^, ^16^, ^17^, ^18^, ^19^ all of which were non‐randomised cohort studies (9 retrospective and 2 prospective). Figure 1 describes the PRISMA flowchart of the inclusion and exclusion of the studies. Six studies were conducted in the United States (US), two in Brazil and one each in the United Kingdom (UK), Spain and Australia. Table 1 depicts the baseline characteristics of the patients included in the studies.

**TABLE 1:** Baseline data of included patients in the systematic review.

Authors	Study type	Year	Total number of patients	Type of SUI surgery performed	Definition of success	Definition of failure
Castle EP	retrospective cohort	2005	38	Bone anchored sling	0–1 pad/day or social continence	assessed with urodynamics ‐ no specific definition
Habashy D	retrospective cohort	2017	80	AdVance sling	NR (pad use pre‐ and post‐surgery compared)	NR
Krughoff K	retrospective cohort	2023	92	Artificial Urinary Sphincter	No need for revision	NR
Lai HH	retrospective cohort	2011	129	Artificial Urinary Sphincter	ICS definitions	ICS definitions
Paiva OG	retrospective cohort	2018	22	Argus T sub‐urethral sling	NR	NR
Serra AC	retrospective cohort	2013	58	AdVance male sling (suburethral)	0 pad; Improvement was defined as a 24 h‐PW reduction of >50%.	All other outcomes were defined as failures
Thiel DD	retrospective cohort	2006	86	Artificial Urinary Sphincter	0–1 pad/day or social continence	>1 pad/day
Toia B	retrospective cohort, multicentre	2021	130	AdVanceXP male sling	0–1 pad/day or 24 h pad weight < 400 g	>1 pad/day or >400 g 24 h pad weight
Warner JN	prospective cohort	2012	38	Transobturator sling	cured (0–1 dry “security” pad) or greatly improved (1–2 pads or pad reduction >50%)	pad reduction <50%, unchanged, or worse SUI, based on daily pad usage by patient history
Zuckerman JM	retrospective cohort	2014	102	AdVance transobturator male slings	cured (0–1 pad) or improved (pad reduction >50% or patient satisfaction)	>1 pad/day or pad reduction <50%, unchanged, or worse SUI
Flavio Trigo Rocha	prospective cohort	2008	40	Artificial Urinary Sphincter	0–1 pad/day or social continence	>1 pad/day or pad reduction <50%, unchanged, or worse SUI
Total number AUS only Sling only			792 347 445			

SUI: Stress Urinary Incontinence; ICS: International Continence Society; NR: Not Recorded.

### 3.2 Patient characteristics

The mean age of the patient cohort was 67.2 (+/− 2.2) years, all were male. Four studies also included men with incontinence after benign bladder outflow surgery in addition to post‐radical prostatectomy incontinence. The data sets did not allow analysis of benign prostatic surgery as a separate subset. All patients underwent urodynamics prior to male incontinence surgery. Patients in seven studies underwent a male sling and the remaining four underwent an artificial urinary sphincter (AUS) insertion for post‐prostatectomy incontinence. The slings used included advance sling (n = 4), bone anchored sling (n = 1), transobturator sling (n = 1), Argus sling (n = 1). On Urodynamics, 29% (n = 229) patients had DO prior to stress incontinence surgery. In one cohort of patients the DO was treated with anticholinergics, the remaining were not treated prior to the incontinence procedure. The authors do not report on the outcome of the anticholingeric treatment prior to surgery. The mean follow‐up period of these patients postoperatively was 34 months (+/−12 months).

### 3.3 Perioperative outcomes

The definition of success of the male incontinence surgery varied among the studies. Seven studies defined success as 0–1 pad/day or achievement of social continence and improvement as 24‐hour pad weight reduction of 50%. Two studies did not report a definition of success while two studies reported definitions of success as standard International Continence Society (ICS) definition and revision surgery not required. Of the studies reporting number of patients with a successful outcome (n = 549), 69% had a successful outcome (n = 377/549).

Three studies did not report a definition of failure, one defined failure “as per ICS definition”, six studies reported failure as use of >1 pad/day or <50% pad reduction and one defined failure as per urodynamics (but no fixed definition). Of the studies reporting failure, 26% (132/499) of patients had reported failure of male incontinence surgery based on the above definitions. Table 2 depicts the outcomes among the patients included in the study.

**TABLE 2:** Outcomes of patients included in the systematic review.

Authors	Preoperative UDS proven DO (number)	Number of patients with successful outcome	Number of patients who failed surgery	Number of DO patients that failed postoperatively	Statistical analysis to ass DO patient outcome	Study conclusion
Castle EP	6	15	23	4	Fisher's exact test to assess statistical differences	Trend to treatment failure with DO
Habashy D	10	15	nr	nr	Multivariate analysis, pairwise comparison assess change in pad use over time	DO adversely affected mid‐term outcome of sling
Krughoff K	27	nr	nr	nr	multivariable cox regression	Earlier surgical intervention (earlier time to implant failure) was associated with DO (HR 1.95, P < 0.01 CI 1.22–3.1). Statistically significant associations for DO (P < 0.01) and pad weight (P = 0.02)
Lai HH	24	nr	nr	nr	t test and Fisher test	Presence of preoperative OAB (with or without DO) does not worsen the overall continence results of the AUS
Paiva OG	10	nr	nr	nr	Fisher, Wilcoxon and Mann–Whitney tests	Surgery resulted in significant reduction of detrusor overactivity
Serra AC	21	49	12	nr	univariate regression	No correlation between the urodynamic findings and surgical outcomes
Thiel DD	34	71	15	6	univariate regression	The presence of DO or preoperative urgency did notcorrelate with the outcome parameters in our study.
Toia B	46	99	31	19	t test and Chi‐squared	incidence of DO in the non‐successful group was significantly higher than in the success group (55% versus 29%, p = 0.009),
Warner JN	9	28	10	3	chi‐squared test	DO had no effect on outcomes
Zuckerman JM	31	64	37	nr	logistic regression	On multivariate Cox regression analysis, DO and an elevated pdetQmax were the only significant factors and both negatively influenced continence outcomesto be predictive of being cured
Flavio Trigo Rocha	10	36	4	nr	t test and fisher test	The presence of DO was not associated with worse continence results (P 0.99) or development of overactive bladder symptoms in the postoperative period (P 0.24).
**Overall**	**228 (29%)**	**377 (69%)**	**132 (26%)**	**32 (34%)**		
**Sling only**	**134 (30%)**	**270 (64%)**	**113 (30%)**	**26 (43%)**		
**AUS only**	**95 (27%)**	**107 (85%)**	**19 (15%)**	**6 (18%)**		

DO: Detrusor Overactivity; nr: Not Recorded.

### 3.4 Sub‐group analysis

Analysing the 4 studies utilising AUS with 347 patients, 27% (n = 95/347) had preoperative DO prior to AUS implantation. The overall postoperative success reported was 85% (n = 107/126) and the failure rate was 15% (n = 19/126).

The remaining 7 studies with 445 patients underwent sling insertion, the preoperative DO was 30% (n = 134/445). The overall postoperative success was 64% (n = 423/445) and failure rate was 30% (n = 373/445).

The difference in the success and failure rates between the two groups was significant statistically (p < 0.001).

### 3.5 Outcomes in patients with DO preoperatively

Owing to the absence of randomised controlled trials, we were unable to perform a meta‐analysis.

We attempted to pool data in studies where raw data was published. The reporting of outcomes in patients with DO preoperatively was variable. Four studies reported number of patients with DO preoperatively that failed the male incontinence surgery postoperatively, the failure rate was reported as 34% (32/95). Despite a higher proportion of men in the group with pre‐operative DO failing the incontinence surgery, the difference was not significant statistically (p = 0.07, chi‐square test). The remaining studies did not publish raw numbers of DO patients that failed stress incontinence surgery but rather published the statistical outcome directly. Only one study group was treated for DO medically prior to male incontinence surgery.

On sub‐group analysis, only one of the four AUS studies reported the failure rate in the patients with preoperative DO as 18% (n = 6/34) while in the sling sub‐group was the failure rate in patients with preoperative DO was 43% (n = 26/61). The difference in the two surgical sub‐groups in terms of preoperative DO and failure rate was statistically significant (p < 0.001).

The statistical method of computing surgical outcomes in patients with preoperative DO on urodynamics was variable. Five studies used regression analysis, of which three used multivariate and two used univariate analysis. The three studies using multivariate regression analysis reported an adverse surgical outcome in men with preoperative DO undergoing stress incontinence surgery. In the two studies using univariate regression analysis, the presence of DO did not influence surgical outcome.

Six studies used Fisher's test, chi‐square test or t‐test to calculate the outcome. In one of these studies, the incidence of DO was significantly higher in the non‐successful group compared to the successful group, one reported a trend to overall treatment failure in patients with preoperative DO and one study reported a reduction in DO in men undergoing stress incontinence surgery. In the remaining three studies preoperative DO was not reported to affect surgical outcomes adversely.

On sub‐group analysis, of the studies on patients who underwent AUS insertion, three studies reported no impact of DO on postoperative continence (univariate analysis and t‐test) while one study with 93 patients reported a worse outcome in patients with preoperative DO (multivariable analysis). In the sling sub‐group, four studies (multivariable regression, t‐test and Fisher's test) reported an adverse impact of DO on postoperative outcomes while the remaining three studies reported no impact (Fisher's test, chi‐square test and univariable regression).

Overall, no conclusion on the effect of preoperative DO in men undergoing stress incontinence surgery can be drawn from the data on account of the variability in definitions of success and failure and variability in statistically analysing the effect of DO on the surgical outcome.

### 3.6 Risk of bias

**FIGURE 2:**
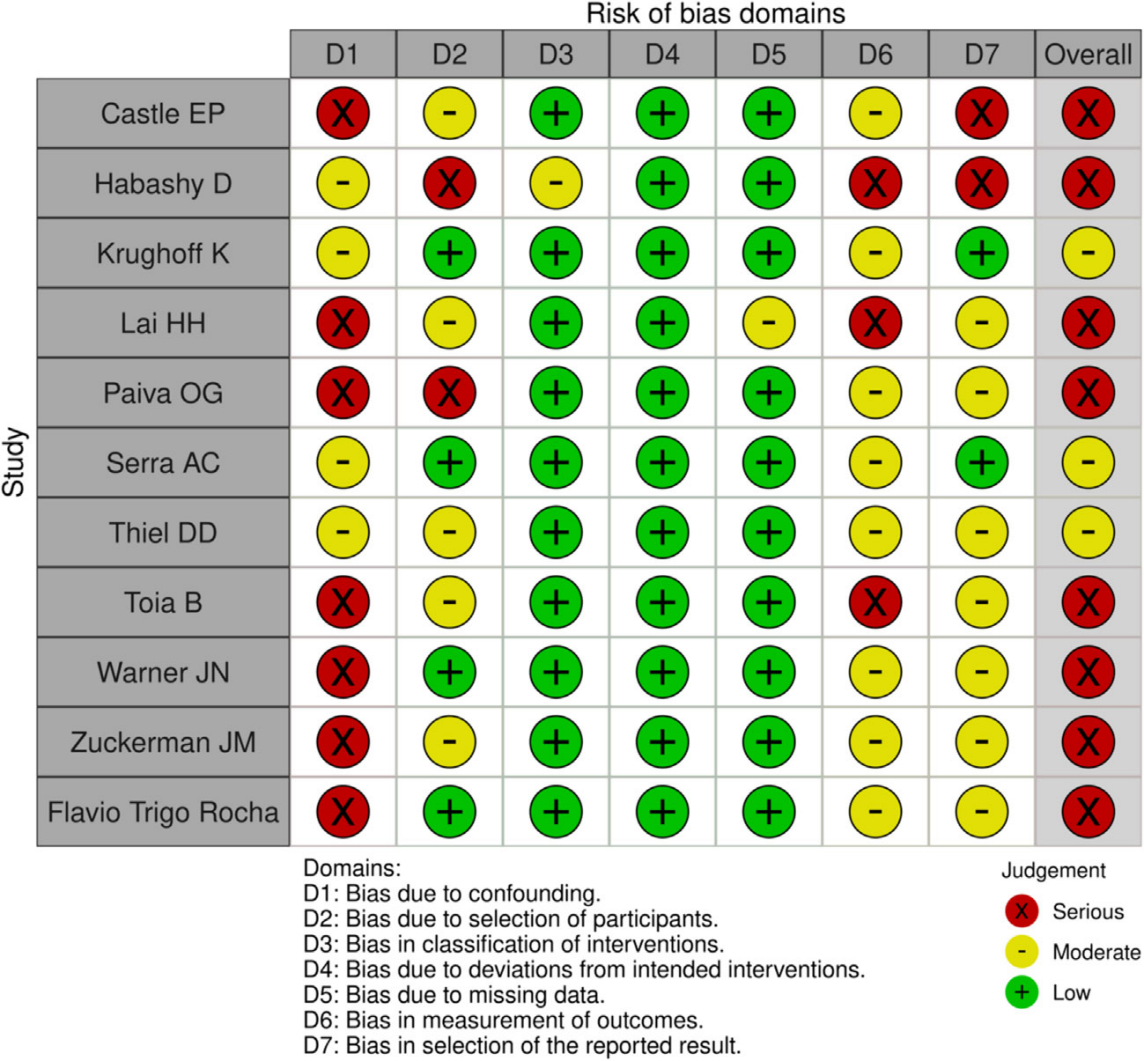
Risk of bias analysis.

The ROBINS‐I tool^20^ was used for risk of bias analysis as all of the studies in this systematic review were non‐randomised cohort studies. A visual representation of the risk of bias analysis is presented in Figure 2. The risk of bias ranges from moderate to serious. None of the studies had a low risk of bias.

## 4 DISCUSSION

To the best of the authors' knowledge this is the first systematic review investigating the outcome of male incontinence surgery in men with preoperative DO. There was significant variation in definitions used for success and failure of male incontinence surgery across the studies included in this systematic review; however, the overall success rate after pooling the raw data was 69% while failure was reported in 26% of patients undergoing male incontinence surgery.

The 2018 American Urological Association guidelines suggest the cure rate for a male sling is approximately 62%, but mention the varying definitions of cure used in 14 papers; there was little evidence found for AUS.^21^ A recent review by Smith et al^22^ also reported variability of definitions to report success after male incontinence surgery with a male sling and AUS remaining the mainstay of treatment in this cohort of patients. This was comparable to our findings; however, slings were used in a higher number of studies than sphincters in this cohort. The most common definition used was social continence (0–1 pad/day) as reported in this systematic review. Success rates of both procedures reported in this review were 70–90%, comparable to the findings of our review. The primary outcome in the MASTER trial was that patients reported incontinence of any frequency greater than “none” or other than “never”, they reported an incontinence rate of 87% in the male sling group and 84% in the sphincter group. The lower cure rate in this study could be secondary to the strict definition used.^23^

On sub‐group analysis, the AUS group had statistically significant higher postoperative success and lower failure rate compared to the sling group, in keeping with the MASTER trial where secondary and post‐hoc analysis were in favour of AUS. In addition, a recent systematic review also reported AUS was better than slings for moderate PPI‐SUI.^24^

Filling cystometry abnormalities (DO and loss of compliance) are present in 31% of men with prostate cancer. Kan et al demonstrated de‐novo storage symptoms in 34% men after radical prostatectomy at 6 months.^7^ De‐novo OAB was found in 44% of patients at 3 months after radical prostatectomy in another study by Lee et al.^21^ These figures are comparable to our cohort. In patients with DO on urodynamics prior to male incontinence surgery, the failure rate was 34% in this systematic review. Hence approximately a third of patients undergoing treatment for prostate cancer (e.g. RP) develop denovo overactive bladder symptoms postoperatively and can have DO on urodynamics that contributes to the urinary symptoms and incontinence after prostate cancer treatment.

Factors influencing the risk of failure or reoperation after male incontinence surgery have been previously studied. Radiotherapy is one of the factors consistently reported to have an adverse impact on the outcome. This was also seen in the MASTER trial^23^ and reported in the European Association of Urology guidelines as a risk factor for urethral atrophy/erosion after AUS insertion.^25^ The AUA guidelines and the ICI (International Consultation on Incontinence) both recommend AUS over a sling in men with previous radiotherapy on the basis of high risk of adverse outcomes postoperatively.^26^, ^27^ However, none of the guidelines make a clear recommendation on the management of patients with DO on urodynamics prior to male incontinence surgery. The ICI^27^ guidelines mention preoperative DO and reduced compliance as causes of poorer intermediate outcome in men with fixed transobturator slings, and suggest urodynamics in men with persistent incontinence after AUS insertion to check for DO or loss of compliance which may be treated separately.

A wide variation was seen in the statistical analyses conducted to calculate the effect of preoperative DO on surgical outcomes in male incontinence surgery. Five studies reported an adverse outcome in men with preoperative DO undergoing male incontinence surgery while the remaining six did not report any effect of DO on the outcome. In our review, the failure rate of male incontinence surgery was higher in the DO group compared to the overall group, but the difference was not statistically significant. Considering the sub‐group analysis, the failure rate with preoperative DO was significantly higher in the sling group (43%) than in the AUS group (18%).

Overall, no definitive conclusion about the effect of DO on male incontinence surgery could be drawn on account of the variability of report success and failure, the differences in outcome reporting and analyses in DO patients. Considering the common occurrence of this finding on urodynamics in men after prostate cancer treatment it remains an important question to be answered. Until then, in the absence of high‐quality evidence, clinicians' judgement and experience are advocated to tailor the management of DO in men eligible for male incontinence surgery on an individual basis.

A limitation of this systematic review pertains to the limitations of the primary included studies, our risk of bias assessment revealed a moderate to serious risk of bias in these non‐randomised, largely retrospective cohort studies. Furthermore, heterogeneity of outcomes limited our ability to pool data in this systematic review to answer the research question.

## 5 CONCLUSIONS

The current optimal management of men with DO on preoperative urodynamics prior to male incontinence surgery is not well studied. We performed a systematic review of the literature including articles on patients undergoing SUI surgery after prostatectomy with preoperative DO. The review was limited by the outcome heterogeneity, variability in study inclusion criteria, reporting and analysis and the quality of the available studies. Within the limitations of the SR, the AUS sub‐group had significantly better postoperative outcomes compared to the sling group in this review.

Over a third of men undergoing male incontinence surgery present with preoperative DO on urodynamics, hence high‐quality studies with standardised definitions and analysis of outcomes are imperative to allow surgeons and patients suffering from DO to better understand the benefits and risks involved with surgery. Within the limitations of the data, this review did not show a statistically significant higher failure rate of male incontinence surgery in patients with DO, although the failure rate was higher in this group. Hence, patients with DO on preoperative urodynamics who are eligible for male incontinence surgery should not be denied surgery but should be counselled appropriately of the risks and potential need for subsequent treatment, to manage expectations.

## AUTHOR CONTRIBUTIONS

Protocol/project development: NB, RD, CI, LS. Data collection or management: NB, SI, AN. Data analysis: NB, SI, AN. Manuscript writing/editing: NB, SI, AN, RD, CI, LS. The authors have no conflict of interest to disclose.

## CONFLICT OF INTEREST STATEMENT

The authors declare no conflict of interest.

## Appendix A Search strategy for systematic review.

Search Strategy: Medline, EMBASE, Emcare, CINAHL, Cochrane (Reviews and Trials), Scopus).

‐‐‐‐‐‐‐‐‐‐‐‐‐‐‐‐‐‐‐‐‐‐‐‐‐‐‐‐‐‐‐‐‐‐‐‐‐‐‐‐‐‐‐‐‐‐‐‐‐‐‐‐‐‐‐‐‐‐‐‐‐‐‐‐‐‐‐‐‐‐‐‐‐‐‐‐‐.

1 Urinary Incontinence, Urge/ or Urinary Incontinence, Stress/ or exp *Urinary Incontinence/or “incontinen*”.mp

2 exp *Prostatectomy/or “prostatectom*”.mp. or “post‐prostatectom*”.mp.

3 1 and 2

4 exp *Urinary Bladder, Overactive/or “overactive detrusor”.mp. or “detrusor overactiv*”.mp.

5 3 and 4

6 limit 5 to (male and humans and yr = “2003–2023” and english).

***************************.
